# Draft genome sequences of *Limosilactobacillus fermentum* BIL20 and BIL24, from Philippine fermented fish, feature biosynthetic gene clusters with diverse potential bioactivities

**DOI:** 10.1128/mra.00075-25

**Published:** 2025-06-18

**Authors:** Joshua T. Veluz, Paul Christian T. Gloria, Maria Auxilia T. Siringan, Irineo J. Dogma,

**Affiliations:** 1 Natural Sciences Research Institute, University of the Philippines-Diliman 54727, Quezon City, Metro Manila, Philippines; 2 The Graduate School, University of Santo Tomas, Manila city, Metro Manila, Philippines; Wellesley College, Wellesley, Massachusetts, USA

**Keywords:** biosynthetic gene clusters, burong isda, lactic acid bacteria, probiotics, whole-genome sequencing

## Abstract

This announcement presents the high-quality draft genomes of *Limosilactobacillus fermentum* BIL20 and BIL24, isolated from Philippine fermented fish, with probiotic potential. The genomes feature biosynthetic gene clusters linked to antibacterial, antifungal, and cytotoxic activities. This announcement contributes to the limited genomic data on lactic acid bacteria from Philippine fermented foods.

## ANNOUNCEMENT

Many lactic acid bacteria (LAB) isolated from Philippine fermented foods exhibit probiotic potential (1, 2), but most studies focus on *in vitro* evaluations. Few reports (3, 4) utilize genomic data, which are essential for accurately identifying and characterizing probiotic LAB (5, 6). This genome announcement adds to the limited genomic data on potential probiotic LAB from Philippine fermented fish. Isolates BIL20 and BIL24 exhibited these probiotic properties, demonstrating bile salt and pH tolerance and producing metabolites that inhibit medically relevant fungal species (7).

The two bacteria were isolated from freshly prepared fermented fish (burong isda) obtained from Arayat, Pampanga, Philippines (15.1650° N, 120.7803° E). The samples were fermented for 10 days at room temperature (8). The samples were homogenized in peptone water (0.1%, wt/vol), diluted up to 10^−5^, and 100 µL was spread-plated on de Man, Rogosa, and Sharpe (MRS) agar (9). Plates were incubated microaerophically at 37°C for 48–72 hours (10, 11). Random circular white colonies were isolated using the t-streak method, incubated under similar conditions, and further purified (10). Both isolates were initially identified via 16S rDNA gene analysis, as previously reported (7). The consensus sequences of BIL20 and BIL24 were deposited in GenBank under accession numbers OQ860095 and OQ860094, respectively. Both isolates were identified as *Limosilactobacillus fermentum*, an identification further supported by the whole genome analysis described below.

The DNA (>100 ng/µL) of 24-hour-old BIL isolates grown in MRS broth under the above-mentioned conditions was extracted using the DNeasy PowerSoil Pro Kit and submitted to Macrogen, Inc. (South Korea). Library preparation was performed using the TruSeq Nano DNA Library Kit and TruSeq Nano DNA Sample Preparation Guide (Part # 15041110 Rev. D) of Illumina. The samples were then sequenced using the Illumina NovaSeq6000 platform with a read length of 151 bp paired-end and 2 Gb throughput. All bioinformatics tools were run by default unless specified otherwise. BIL20 and BIL24 raw sequences contained 3,384,475,948 total bases with 22,413,748 total reads (50.8% G + C) and 2,058,396,364 total bases with 13,631,764 total reads (50.6% G + C), respectively. The raw reads were Q30-trimmed using Trimmomatic version 0.36 (12) and assembled with SPAdes version July 2023 (13) through the Unicycler version 0.5 (14) tool using normal bridging mode. The characteristics and identity of the genome assemblies are presented in Table 1. The two isolates exhibited highly similar genome characteristics yet were determined to be distinct at the strain level (7).

**TABLE 1:** Genome characteristics and taxonomic identity of the BIL assemblies

Parameters		BIL20	BIL24
Genome accession No.		GCA_037168225.1	GCA_037164145.1
CheckM analysis (version 1.2.3) (15)	Completeness (%)	90.57	90.57
	Contamination (%)	4.5	4.5
BUSCO completeness (%, version 5.8.0) (16)		91.8	91.80
Genome size (bp)		1,989,813	1,991,527
Number of contigs		238	238
*N*_50_ (bp)		28,017	28,480
*L*_50_		23	23
GC (%)		52.05	52.05
Genome coverage		842	414
Genome Taxonomy Database (GTDB, version 2.3.2) (17) identity		*Limosilactobacillus fermentum*	*Limosilactobacillus fermentum*

The BIL20 and BIL24 genomes were annotated using NCBI’s Prokaryotic Genome Annotation Pipeline version 6.9 (18), predicting 2,803 genes (1,906 protein coding) and 2,807 genes (1,903 protein coding), respectively. The two genomes were also subjected to DeepBGC version 0.1.31 (19). Biosynthetic gene clusters (BGCs) were predicted in the genomes, with BIL20 and BIL24 containing 59 and 56 BGCs, respectively. These included nonribosomal peptides (NRPs), polyketides, ribosomally synthesized and post-translationally modified peptides (RiPPs), and saccharides. The BIL20 genome exhibited 52, 3, and 3 BGCs associated with antibacterial, antifungal, and cytotoxic activity, respectively. The BIL24 genome was predicted to contain 49, 2, and 3 BGCs with potential antibacterial, antifungal, and cytotoxic activity, respectively.

## Data Availability

The BIL raw reads (SRX25010227 and SRX25010228) and assemblies (GCA_037168225.1 and GCA_037164145.1) were deposited in NCBI BioProject PRJNA1077654. All data featured in this genome announcement, including the DeepBGC results, can be accessed at zenodo.org/records/14636720.

## References

[1] Agcaoili GJT, Oyong G, Cabrera EC. 2019. Screening of lactic acid bacteria isolated from Philippine traditional fermented products: potential probiotic bacteria with antimicrobial and cytotoxic activities. The FASEB Journal 33. doi:10.1096/fasebj.2019.33.1_supplement.38.9

[2] Navarro RR, Faronilo KML, Eom S-H, Jeon K-H. 2018. Isolation, characterization, and identification of probiotic lactic acid bacterium from Sabeng, a Philippine fermented drink. J Int Soc Southeast Asian Agric Sci 24:127–136.

[3] Lara ZB, Amoranto MBC, Elegado FB, Dalmacio LMM, Balolong MP. 2024. Draft genome sequence of Pediococcus acidilactici 3G3 isolated from Philippine fermented pork. Microbiol Resour Announc 13:e0129923. doi:10.1128/mra.01299-23 38526097 PMC11008191

[4] Lara ZB, Amoranto MBC, Elegado FB, Dalmacio LMM, Balolong MP. 2024. Draft genome sequence of Lactiplantibacillus plantarum BS25 isolated from a local fermented rice-shrimp mixture from the Philippines. Microbiol Resour Announc 13:e0080823. doi:10.1128/mra.00808-23 38179907 PMC10868257

[5] Ventura M, Turroni F, van Sinderen D. 2012. Probiogenomics as a tool to obtain genetic insights into adaptation of probiotic bacteria to the human gut. Bioengineered 3:73–79. doi:10.4161/bbug.18540 PMC3357336 22095053

[6] Bambury A, Sandhu K, Cryan JF, Dinan TG. 2018. Finding the needle in the haystack: systematic identification of psychobiotics. Br J Pharmacol 175:4430–4438. doi:10.1111/bph.14127 29243233 PMC6255950

[7] Veluz JT, Gloria PCT, Siringan MAT, Dogma IJ. 2025. Beyond Buro: genomics and assays revealed the probiotic and antifungal traits of Limosilactobacillus fermentum from Philippine burong isda (fermented fish). The Microbe 7:100334. doi:10.1016/j.microb.2025.100334

[8] Olympia M, Ono H, Shinmyo A, Takano M. 1992. Lactic acid bacteria in fermented fishery product, “burong bangus”. Journal of Fermentation and Bioengineering 73:193–197. doi:10.1016/0922-338X(92)90159-R

[9] Bulgasem BY, Lani MN, Hassan Z, Wan Yusoff WM, Fnaish SG. 2016. Antifungal activity of lactic acid bacteria strains isolated from natural honey against pathogenic Candida species. Mycobiology 44:302–309. doi:10.5941/MYCO.2016.44.4.302 28154488 PMC5287163

[10] Bin Masalam MS, Bahieldin A, Alharbi MG, Al-Masaudi S, Al-Jaouni SK, Harakeh SM, Al-Hindi RR. 2018. Isolation, molecular characterization and probiotic potential of lactic acid bacteria in Saudi raw and fermented milk. Evid Based Complement Alternat Med 2018:7970463. doi:10.1155/2018/7970463 30147735 PMC6083559

[11] Elbanna K, El Hadad S, Assaeedi A, Aldahlawi A, Khider M, Alhebshi A. 2018. In vitro and in vivo evidences for innate immune stimulators lactic acid bacterial starters isolated from fermented camel dairy products. Sci Rep 8:12553. doi:10.1038/s41598-018-31006-3 30135492 PMC6105719

[12] Bolger AM, Lohse M, Usadel B. 2014. Trimmomatic: a flexible trimmer for Illumina sequence data. Bioinformatics 30:2114–2120. doi:10.1093/bioinformatics/btu170 24695404 PMC4103590

[13] Bankevich A, Nurk S, Antipov D, Gurevich AA, Dvorkin M, Kulikov AS, Lesin VM, Nikolenko SI, Pham S, Prjibelski AD, Pyshkin AV, Sirotkin AV, Vyahhi N, Tesler G, Alekseyev MA, Pevzner PA. 2012. SPAdes: a new genome assembly algorithm and its applications to single-cell sequencing. J Comput Biol 19:455–477. doi:10.1089/cmb.2012.0021 22506599 PMC3342519

[14] Wick RR, Judd LM, Gorrie CL, Holt KE. 2017. Unicycler: resolving bacterial genome assemblies from short and long sequencing reads. PLOS Comput Biol 13:e1005595. doi:10.1371/journal.pcbi.1005595 28594827 PMC5481147

[15] Parks DH, Imelfort M, Skennerton CT, Hugenholtz P, Tyson GW. 2015. CheckM: assessing the quality of microbial genomes recovered from isolates, single cells, and metagenomes. Genome Res 25:1043–1055. doi:10.1101/gr.186072.114 25977477 PMC4484387

[16] Simão FA, Waterhouse RM, Ioannidis P, Kriventseva EV, Zdobnov EM. 2015. BUSCO: assessing genome assembly and annotation completeness with single-copy orthologs. Bioinformatics 31:3210–3212. doi:10.1093/bioinformatics/btv351 26059717

[17] Chaumeil P-A, Mussig AJ, Hugenholtz P, Parks DH. 2020. GTDB-Tk: a toolkit to classify genomes with the genome taxonomy database. Bioinformatics 36:1925–1927. doi:10.1093/bioinformatics/btz848 PMC7703759 31730192

[18] Tatusova T, DiCuccio M, Badretdin A, Chetvernin V, Nawrocki EP, Zaslavsky L, Lomsadze A, Pruitt KD, Borodovsky M, Ostell J. 2016. NCBI prokaryotic genome annotation pipeline. Nucleic Acids Res 44:6614–6624. doi:10.1093/nar/gkw569 27342282 PMC5001611

[19] Liu M, Li Y, Li H. 2022. Deep learning to predict the biosynthetic gene clusters in bacterial genomes. J Mol Biol 434:167597. doi:10.1016/j.jmb.2022.167597 35526560

